# A Conversation with Matthias Rillig, Soil Ecologist

**DOI:** 10.1021/acscentsci.5c00813

**Published:** 2025-05-14

**Authors:** Katarina Zimmer

## Abstract

Rillig uncovers how microplastics are transforming soil.

## Graphical abstract

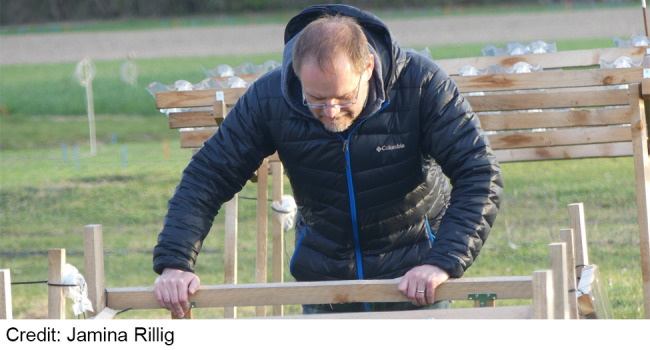

When we think of plastic pollution, our minds often go to trash-smothered oceans or photos of marine life tangled in nets. But scientists are now learning that plastics are also permeating the world’s soils and having noticeable effects on plant and animal health.

Matthias Rillig, an ecologist at the Free University of Berlin, was one of the first scientists to dig into soil and study microplastics, the particles less than 5 mm in length that often result from plastic slowly degrading.

Rillig and his colleagues are uncovering a range of troubling impacts of soil’s so-called plastisphere, such as changes to soil’s ability to store planet-warming carbon and a decline in the health of earthworms and other soil-dwelling animals. Researchers have recently begun turning their attention to bacterial communities that gather around soil microplastics and that have possible knock-on effects for human health.

**Figure:**
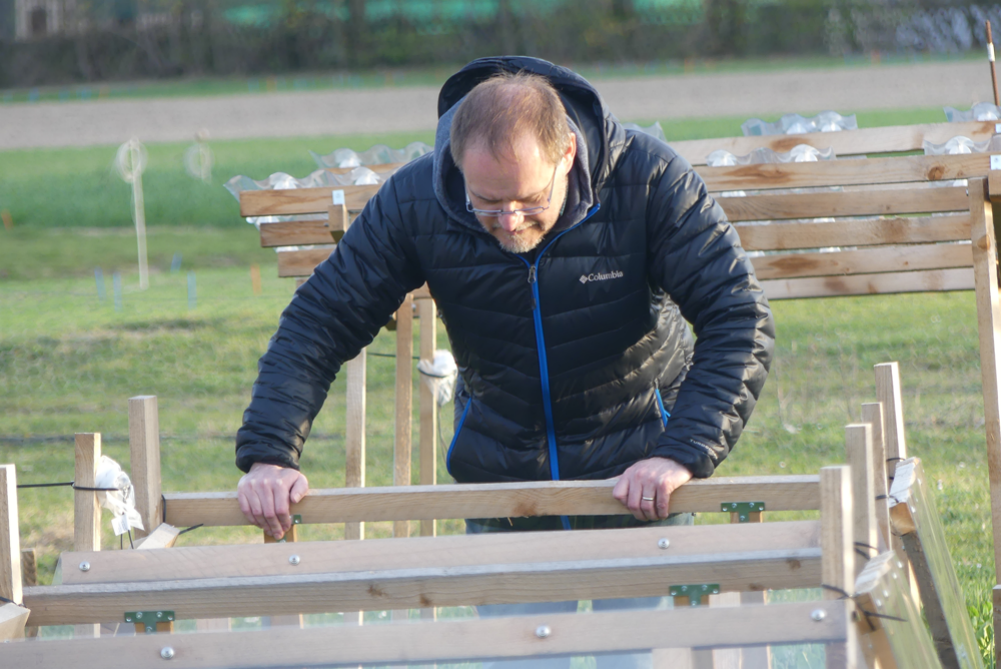
Matthias Rillig checks in on the “Berlin Global Change Experiment.” The research tested the effects of different environmental changes, like warming and drought, on local soils. The results were published in 2024. Credit: Jamina Rillig.

Katarina Zimmer spoke with Rillig about the main impacts of soil microplastics on ecosystems, agriculture, and people, as well as what can be done about them. This interview was edited for length and clarity.

## What are the most important impacts on soil life that we know of so far?

There are three major impacts, starting with physical effects. As opposed to most other chemical pollutants, microplastics are actually particles. Their shape matters. If you have a plastic fiber, that has completely different effects than a fragment, a sphere, or a film. For instance, when you add fibers to the soil, it creates more space in between clump-like aggregates of soil particles, making the soil fluffier and easier for roots to penetrate—sometimes having positive growth effects on plants.

And if you change the physical makeup of soil, you also change how carbon is stored, because it is locked up physically inside soil aggregates. If microplastics make these aggregates less stable, as fibrous microplastics can do, there’s increased fluxes of carbon from soils into the atmosphere.

**Figure:**
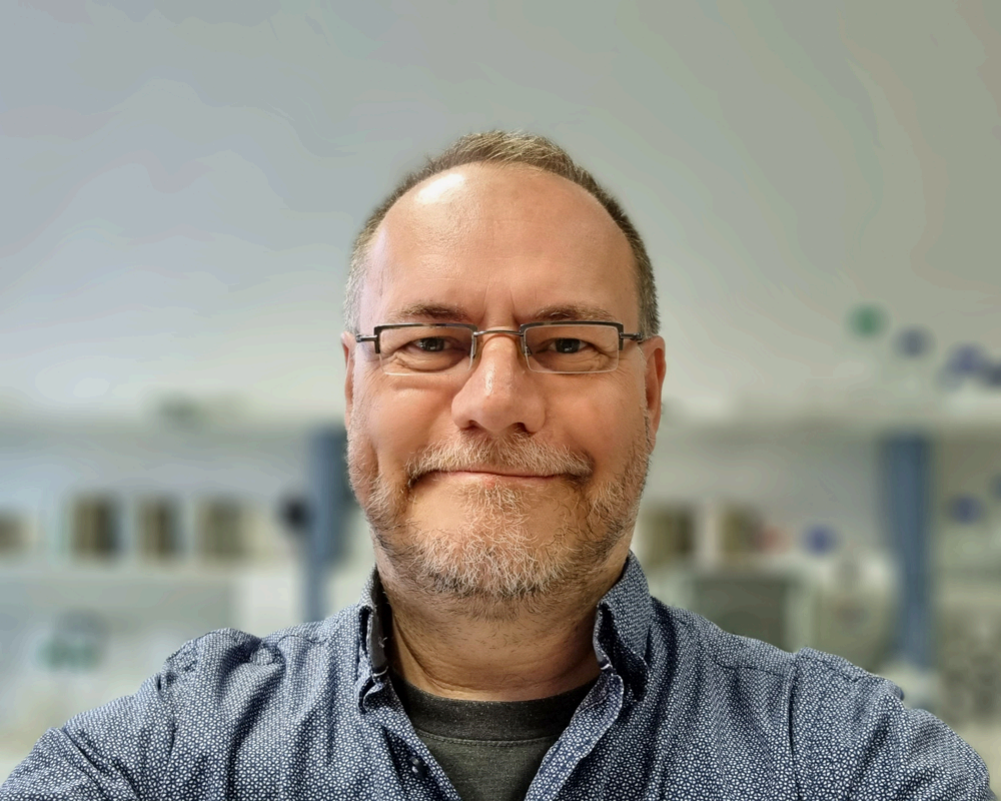
Matthias Rillig. Credit: Courtesy of Mattias Rillig.

The second pathway is chemical toxicity. The problem is not so much the plastic polymers themselves, which are not toxic, but that chemicals leach out of the particles—chemicals that were added to give plastics color, to make them more malleable, or as flame retardants.

In a rather astounding experiment, we exposed nematode worms to different types of microplastics. They were toxic; the nematodes had fewer eggs and offspring. When we washed those particles and gave them to the nematodes again, they were no longer toxic because we had removed the chemicals on the surface that were about to diffuse out. But when you let these freshly washed particles sit around for a week, they become toxic again as more additives leach out.

These particles are a reservoir of toxic compounds that are continuously released, probably over long periods of time. That has propelled us to propose that maybe we have been incurring microplastic toxicity debt. We have committed ourselves to this toxicity, and maybe it has not all been released yet.

Third—and this is the newest of the three mechanisms—microplastic particle surfaces and the soil around them become enriched in certain microbes, especially harmful bacterial pathogens and bacteria with antibiotic resistance genes. If these antibiotic-resistant bacteria somehow get into hospitals and into patients battling infections, they could render antibiotics ineffective.

But this field is so new. My first paper on the topic was 2012, and maybe the first experimental work was 2017, so this research field is still a baby. We do not know the long-term effects very well.

**Figure:**
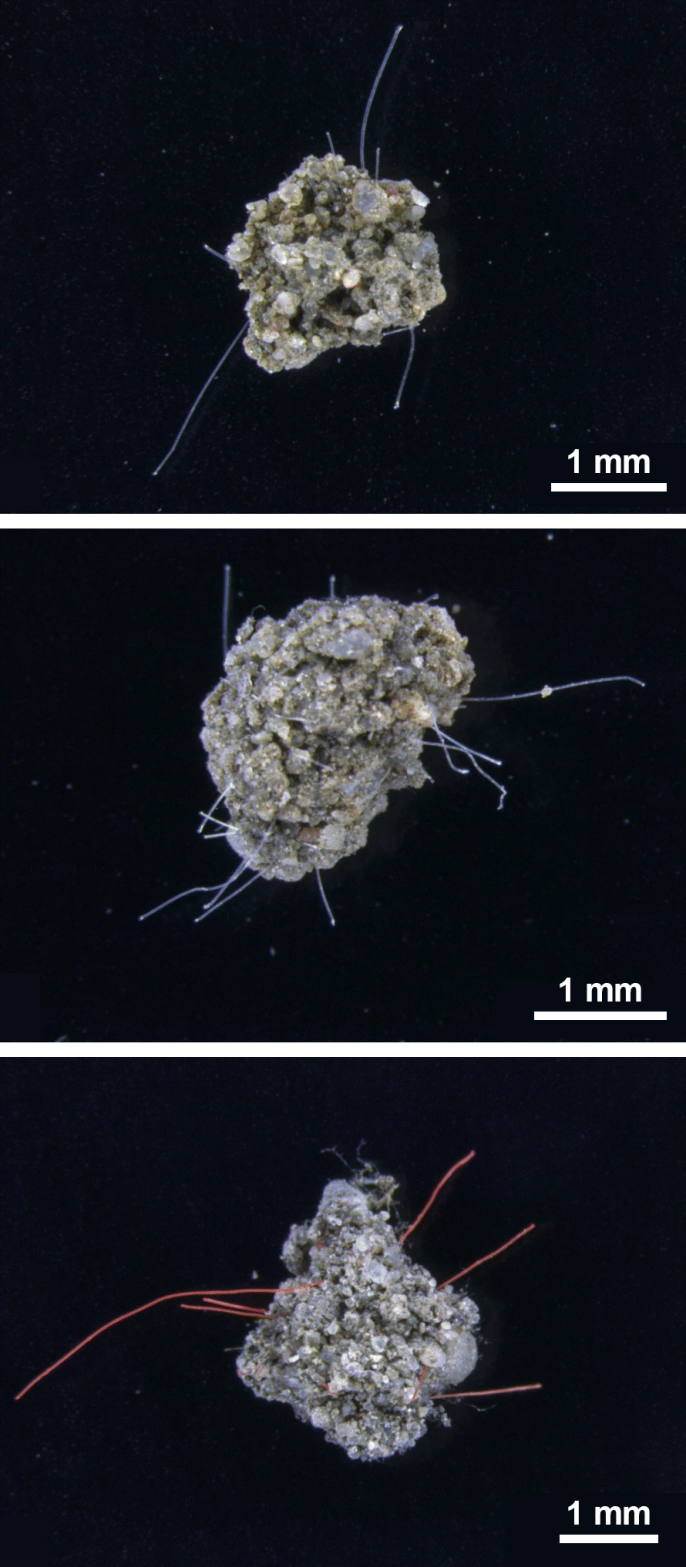
In a 2021 paper, Matthias Rillig’s team assessed how polyamide (from top), polyester, and polypropylene fibers change how soil aggregates. Credit: *Microplast. Nanoplast*.

## What have you learned about the impacts of microplastics on plants?

In an experiment with winter wheat, we tested three different agriculturally important soils from Germany and 10 different microplastic types. The astonishing thing is that we found completely different effects in these different soils.

In one soil type, the effect was more or less neutral. In another soil, growth was reduced, so you may need to compensate by fertilizing more, which is not always sustainable. In the third soil, the effects of the different microplastics were, on average, positive, increasing the plant’s growth.

But I would like to add that a positive response in ecology does not mean it is desirable. We did an experiment with plant communities, and we still saw positive effects on overall productivity. But it was more toward the species that tended to have more of an invasive character.

We’re now trying to dissect which soil properties were responsible for these rather different effects.

## Could soil microplastics in agriculture affect our own plastic exposure?

There’s no doubt that we’re exposed to plastics all the time, including plastics from the soil. Lighter particles in soil get entrained in wind and become airborne. They could get transported to places where they could be inhaled by people or settle on crops that people then eat, for instance.

Another route is when the microparticles are very small or in the nanosize range, they get taken up into plant roots—or by plant leaves, which take them up from the air—and get redistributed around the plant, probably including parts that we eat. Also, when stuff gradually drops down from the atmosphere or is dragged down by rain, it can settle on vegetable leaves, so you might also ingest it if you do not wash the leaves.

## How do microplastics get onto and into the soil, and how much of them are there?

It depends on where you are. If you are standing in a very remote region somewhere in North America, for example, it will mostly get there by stuff raining out of the atmosphere.

But especially in some regions of China, plastic mulching film is used to cover the soil surface to keep it warm and moist. The film they’ve been using is very thin, and it just kind of melts at the end of the growing season and can only be very ineffectively retrieved. The stuff does not decompose readily; it just fragments into smaller and smaller pieces. In such a soil, you basically see microplastic everywhere.

Also, some fertilizers are coated with polymers. Plastic also gets in with irrigation tubing or with plastic netting that protects crops.

How much of it there is is not easy to say. The detection and analysis of soil microplastic is very challenging. Estimates range from almost zero or a few dozen particles per kilogram of soil to hundreds of thousands or millions of particles.

## How can we adjust agricultural practices to avoid these kinds of impacts?

Well, “avoid” is maybe—

## Reduce?

“Reduce” is maybe better. “Avoid” is difficult because plastic-free agriculture, I think, is currently not realistic.

There are certain things you can do. One example is that you should not use that thin mulching film; in China there are now laws that require the use of thicker films. And I would not recommend using plastic-coated fertilizers.

But if plastics rain down from the atmosphere, what are you going to do? You are always going to have that input, at least for the foreseeable future.

We recently used data from a field in Rothamsted, UK, to estimate that 50 years out, with business as usual, that soil will have an exponential growth rate of plastic particle numbers—because these particles do not decompose and we’re always adding new ones.

The challenge for the chemical industry is to propose new substances that are completely mineralizable once they get into the environment. The way plastics are being used now—designed for a single use yet lasting hundreds of years or more—is of course not good.

*Katarina Zimmer is a freelance contributor to* Chemical & Engineering News, *an independent news publication of the American Chemical Society*.

